# A continuum approach to model neurites/dendrites with emerging subtrees

**DOI:** 10.1186/1471-2202-14-S1-P73

**Published:** 2013-07-08

**Authors:** Sandra D Berger, Steven M Baer, Sharon M Crook

**Affiliations:** 1 School of Life Sciences, Arizona State University, Tempe, AZ 85287, USA; 2 School of Mathematical and Statistical Sciences, Arizona State University, Tempe, AZ 85287, USA

## 

Neuronal morphology plays an important role in the processing of spatiotemporal synaptic input patterns.

We formulate a new cable theoretic model to study the output in a dendritic cable with many subtrees, both analytically and computationally. The model is an extension of the continuum spine model derived by Baer and Rinzel [1] and like that model, there is no direct interaction between branches. Instead, all interactions occur through the primary dendrite. The continuum branch formulation provides a link between analytical and computational approaches and allows for detailed models of structural plasticity with the advantage that parameters like branch density can be varied dynamically. This is in contrast to multicompartment modeling approaches where different models, each having a static morphology, must be used.

A multicompartment model with equally spaced discrete branches implemented in NEURON [2] is used to validate the continuum branch model. Then we demonstrate the approach using Drosophila Motoneuron 5 (MN5), a monopolar neuron, where soma and axon are connected via a long primary neurite from which dendritic subtrees emerge. Parameters for the model are based on 3D reconstructions [3] of that neuron and the subtrees are reduced to uniform cables.

For a passive membrane and uniformly distributed branches, this formulation provides analytic estimates of electrical parameters, such as the input resistance. Transforming the equations to the frequency domain, we examine the steady state response properties for periodic forcing, where the impact of structural properties on transfer properties and the phase shift at different locations are calculated directly. Based on calculations of the voltage response along the primary neurite, the continuum formulation performs much better than a simple cable as an approximation of the morphologically realistic model. Simulations of the continuum model are in good agreement with the realistic model for frequencies up to 1000 Hz.

In insects neurons the spike initiation zone (SIZ) is usually located far away from the recording site. We use this model to investigate the impact of the location of the SIZ and morphology on spike initiation and spike shape at the recording site as well as on activation and inactivation curves of ion channels obtained by voltage clamp recordings.

Also we investigate how structural properties impact the results found in a point model of MN5, where variations of ion channel densities in physiological ranges lead to different observed firing properties [4].
